# Rin*maker*: a fast, versatile and reliable tool to determine residue interaction networks in proteins

**DOI:** 10.1186/s12859-023-05466-y

**Published:** 2023-09-11

**Authors:** Alvise Spanò, Lorenzo Fanton, Davide Pizzolato, Jacopo Moi, Francesco Vinci, Alberto Pesce, Cedrix J. Dongmo Foumthuim, Achille Giacometti, Marta Simeoni

**Affiliations:** 1 Department of Environmental Science, Computer Science and Statistics, University Ca’ Foscari of Venice, Via Torino 155, 30172 Venice, Italy; 2 Department of Molecular Science and Nanosystems, University Ca’ Foscari of Venice, Via Torino 155, 30172 Venice, Italy; 3 European Centre for Living Technology (ECLT), Dorsoduro 3246, 30123 Venice, Italy

**Keywords:** Protein 3D structure, Residue interaction network (RIN), Non-covalent bonds

## Abstract

**Background:**

Residue Interaction Networks (RINs) map the crystallographic description of a protein into a graph, where amino acids are represented as nodes and non-covalent bonds as edges. Determination and visualization of a protein as a RIN provides insights on the topological properties (and hence their related biological functions) of large proteins without dealing with the full complexity of the three-dimensional description, and hence it represents an invaluable tool of modern bioinformatics.

**Results:**

We present RIN*maker*, a fast, flexible, and powerful tool for determining and visualizing RINs that include all standard non-covalent interactions. RIN*maker* is offered as a cross-platform and open source software that can be used either as a command-line tool or through a web application or a web API service. We benchmark its efficiency against the main alternatives and provide explicit tests to show its performance and its correctness.

**Conclusions:**

RIN*maker* is designed to be fully customizable, from a simple and handy support for experimental research to a sophisticated computational tool that can be embedded into a large computational pipeline. Hence, it paves the way to bridge the gap between data-driven/machine learning approaches and numerical simulations of simple, physically motivated, models.

**Supplementary Information:**

The online version contains supplementary material available at 10.1186/s12859-023-05466-y.

## Background

Recent successes of Alphafold [1] and following up analyses [2, 3] have clearly put in the limelight the power of data driven approaches in structural biology, with the number of predicted folds that is now exceeding the number of experimentally accessible protein crystals by few orders of magnitude. A detailed analysis of the three dimensional structure of a protein is clearly of paramount importance for mapping out its specific properties and hence its functionality. However, a direct use of the three-dimensional structure, when possible, has proven highly computational demanding. Residue Interaction Network (RIN) is a graph where nodes are amino acids and edges represent non-covalent interactions and can be regarded as a possible alternative whenever only information regarding the protein topology and the distribution of the specific non-covalent interactions are required.

While there are various tools in the literature for implementing and analysing RINs—see [4] for a recent survey, most of them are either embedded in a Molecular Dynamics (MD) package or are specifically designed to analyze MD trajectories and to produce as output an analysis of the corresponding networks. Usually such tools determine the $C_{\alpha }$ or $C_{\beta }$ contact maps, or general non-covalent contacts based on atoms proximity. Some tools in the literature calculate specific non-covalent bonds [5–8], but they all come with their own shortcomings and drawbacks. RING 3.0 [5] and RIP-MD [9] are offered as a black box tool as their code is not available. Arpeggio [8] and its recent refactoring PDBe-Arpeggio [10] is an open source tool that considers a wide set of specific bonds. However, the user cannot tune the bonds thresholds, energies are not computed, and the execution is not computationally efficient. In general, a serious drawback of the tools in the literature is that the correctness of the obtained RINs is not addressed. Another common issue concerns the computational efficiency of the RIN calculation: usually the performance evaluation of the proposed algorithm is missing or insufficiently addressed.

A recent study by some of us developed a computational pipeline combining homology modeling, machine learning and graph theory techniques for the analysis of point mutations in the sequence of a specific membrane protein [11]. In that framework, one of the available tools [7] was used to generate the RIN, and that experience prompted the need for a new tool able to overcome all the above drawbacks.

To fill this gap we present RIN*maker*, a tool for determining and visualizing the RIN of a protein given in input. RIN*maker* is offered as a cross-platform and open source software that can be used either as a command-line tool or through a web application featuring a modern 3D user interface. Additionally, we offer a web API service for embedding RIN*maker* into third-party clients. RIN*maker* allows for determining the $C_{\alpha }$ contact map and a comprehensive set of non-covalent interactions. For each considered bond either default or user specified thresholds can be used for the calculation, resulting in a versatile tool. Moreover, we developed a custom integration test system to ensure the program correctness. The whole test suite is distributed along with the application, allowing anyone to reproduce the test results. Finally, computational efficiency was the main focus while designing and developing RIN*maker*. For this reason, we assessed the tool’s efficiency through a series of performance evaluation tests, which highlighted that RIN*maker* can be used in any context where high-throughput production of RINs is required. RIN*maker* is supported by a detailed supplementary document (see Additional file 1) for all employed rules to identify different non-covalent interactions, as well as for the custom test cases used to assess the program correctness.

## Implementation

The main goal in designing RIN*maker* was computational efficiency, which guided all the implementation choices. The style of Generic Programming (GP) [12] has been adopted as it enforces a number of optimizations. By properly defining copy-constructors, assignment operators and move-constructors for custom datatypes, C++ and GP allows avoiding unwanted replications of data structures, boosting the overall performance. By using the template system rather than the classic object-oriented programming style based on subtype polymorphism and dynamic dispatching [13], the C++ language grants maximum efficiency thanks to the static dispatching of methods and to a number of other compile-time optimizations.

A second goal was to ensure the RIN*maker* correctness. To this aim, the use of advanced preprocessor macros and C++11 lambdas played an important role in designing a programmable and versatile test suite.

In this section we present the RIN*maker* workflow and main features and describe how we addressed the efficiency and correctness issues.

### RIN*maker* workflow and main features

**Fig. 1:**
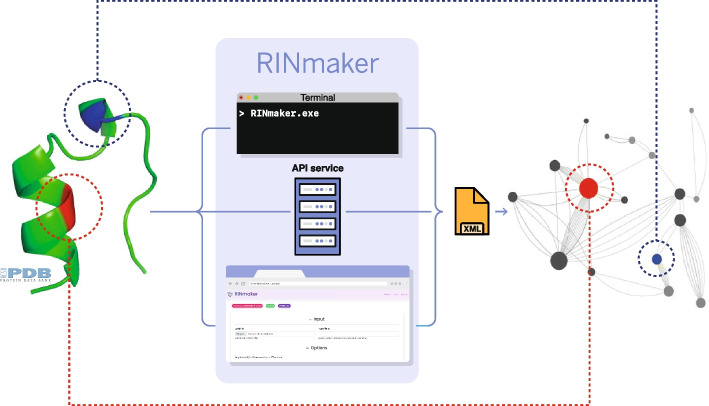
The RIN*maker* flow: The 3D structure of a protein (left) is mapped into a RIN network (right) via different possibilities (command-line, web API and web GUI). As a specific example, the red and blue amino acids highlighted in the protein structure on the left are mapped into the corresponding nodes on the right

**Fig. 2:**
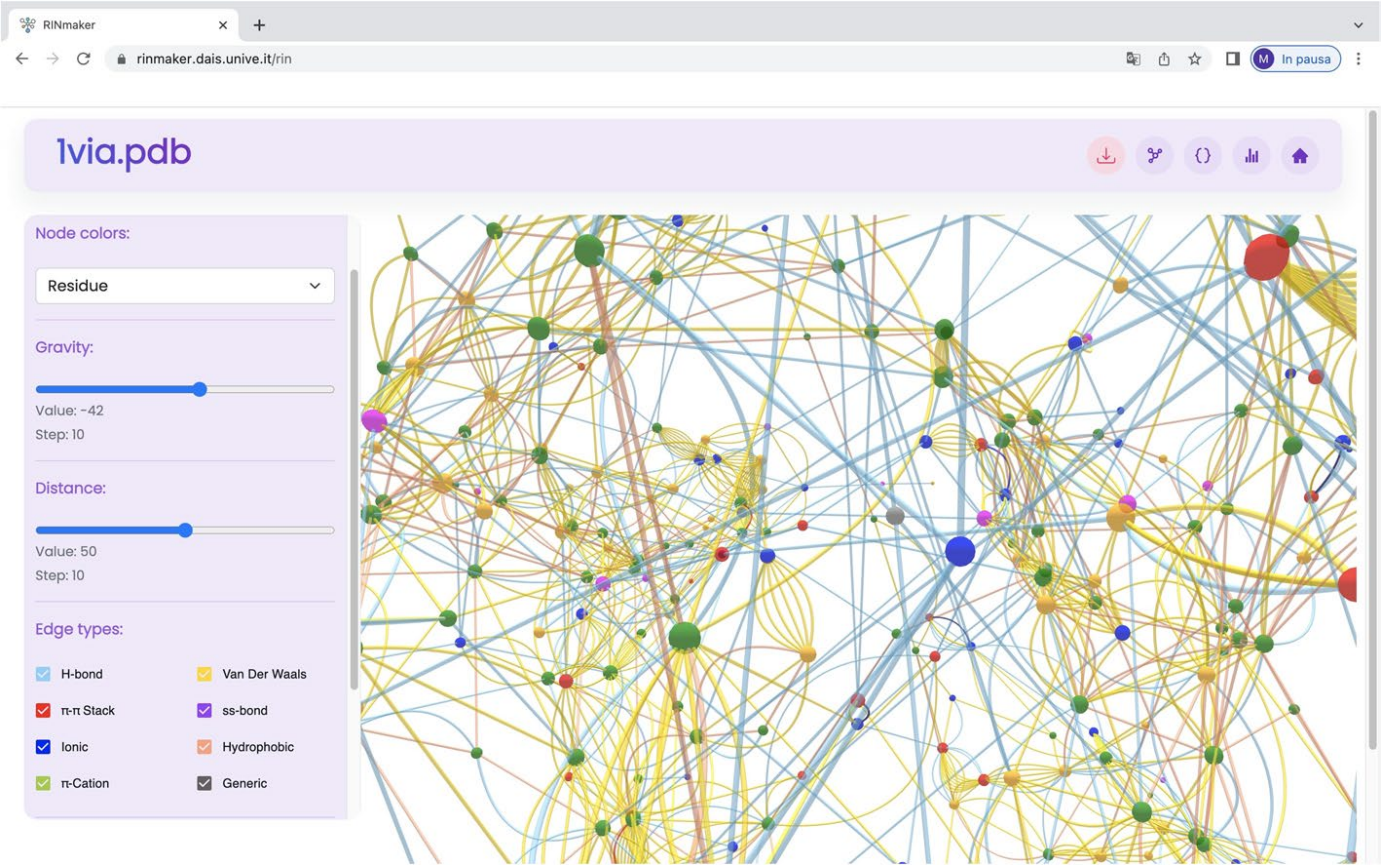
The RIN*maker* user interface: RIN 3D visualization

The RIN*maker* workflow is illustrated in Fig. 1. The core of RIN*maker* consists of one executable that receives in input the 3D structure of a protein and produces in output a file representing the corresponding RIN. RIN*maker* is offered as an application supporting multiple user interfaces:

- A console application from the command line with a wide range of possible arguments allowing the user to fine-tune bond parameters, such as distance threshold, orientational angles, etc. All such parameters have default values that are reasonable in most cases.
- An HTTP-based API available for third-party applications requiring RIN*maker* as a service. Clients can upload an input file through an HTTP post to the API where the content of the input file appears in the body in JSON format and download the resulting RIN via marshalling and unmarshalling in JSON format.
- A GUI web application running RIN*maker* in the background on the server side. The user can search an input file in the PDB databank or upload a local file, select the desired parameters for the non-covalent bonds and visualize the resulting RIN through a real-time rendered 2D or 3D representation, see an example in Fig. 2. Smooth motion, rotation and zoom are available, delivering a 60+ fps experience on an average machine (an Intel i5 10th generation processor with an integrated GPU). This helps the user exploring the RIN, observing bonds and retrieving relevant information on the network structure, the details of nodes and edges and some overall statistics.

The main features of RIN*maker* are the following:

- It supports both mmCIF and PDB as input file formats.
- The output RIN is by default generated in GraphML format [14]. A special CLI option allows for the generation of two CSV output files, one for the nodes and one of the edges of the RIN, in place of a single GraphML file.
- It allows for easily processing snapshots in MD trajectories. The entire MD trajectory, stored into a single PDB file, can be handled by RINmaker to output a single RIN for each individual snapshot. Indeed, RIN*maker* supports multiple models within a single input file. A special command line flag makes RIN*maker* perform a separate run for each model, outputting all the RINs as distinct files into a single directory.
- It determines the $C_\alpha$ contact-map as well as the following non-covalent interactions: H-bonds, Van der Waals interactions, ionic bridges, $\pi$-$\pi$ stacks, $\pi$-cation interactions. We included also “hydrophobic interactions” in view of their frequent reference in the literature [15], even if, strictly speaking, such bond does not exist as it is the effective combination of several different contributions, partially of entropic origin.
- It provides a set of attributes for the RIN nodes and edges, such as the type of bond, the involved atoms, their distance, orientation and energy. For each bond, energy is calculated as a function of the distance between atoms and their relative orientation in space, rather than using approximated constant values available in the literature. This allows for a more accurate evaluation of the interactions. Specific formulas and tables for energy calculation are available in the Additional file 1.

### Computational efficiency

Computational efficiency of RIN*maker* deserves a detailed discussion. Searching for non-covalent bonds among all the atoms of a protein would require $O(n^2)$ operations, *n* being the number of atoms. As a consequence, the choice of an efficient data structure was mandatory. We resorted to the use of kD-Trees, as they allow for an efficient representation of k-dimensional spatial information and a low cost proximity search algorithm [16]. We implemented a kD-Tree library in C++ for achieving maximum performance while retaining a strongly typed approach: dimension *k* is represented by an integer template argument, in the style of template meta-programming [17], which allows for inlining of constant expressions and other compile-time optimizations for $k = 3$, since the protein structure is represented in a 3D space [18]. The program builds a balanced kD-Tree representing the protein 3D structure in $O(n \log ^2 n)$ time, with *n* being the number of atoms in the input file. With such data structure, the search for non-covalent bonds becomes the search for neighbouring nodes in the balanced kD-tree, which runs in $O(\log n)$ time on average and in *O*(*n*) time in the worst case. The advantage of adopting the kD-tree data structure and the related proximity search algorithm clearly emerges when assessing the RIN*maker* performance, as reported in the Results and Discussion section.

### Test suite

RIN*maker* is provided with a custom integration test system based on the Google Test Framework [19]. The program can be compiled in *test* mode to run a series of tests. Tests are on two levels: tests of single code portions and functions; and tests of the whole program over a given input file. Code-level tests are modular and reusable thanks to advanced macro usage within C++, allowing for easy creation of new tests. A series of input sample PDB files specifically designed for testing the program behaviour on the various bonds are included with the RIN*maker* distribution. Moreover, a detailed description of all molecules included in tests along with the expected results is included in the Additional file 1.

RIN*maker* can be built either in *application mode* for producing the normal executable, or in *test mode* for producing a special executable aimed at testing the program. The *test mode* executable can be obtained by compiling the program with a specific compile-time flag, or, more simply, by running *cmake* with the *Test* target.

The *test mode* executable simulates multiple runs of the normal *application mode* execution. This allows for performing a series of tests in a single run of the program. Each test is performed with a specific combination of command line arguments. Moreover, to speed-up the testing process, each output RIN is kept in memory rather than being dumped to an output file, thus avoiding the re-parsing of the output files when comparing the output bonds against the expected ones.

The main advantages of such test system are scalability and maintainability. Writing new test cases with new molecules is straightforward as well as adapting existing ones to new scenarios.

## Results and discussion

In this section we present a case study where RIN*maker* is employed for the analysis of a molecular dynamic trajectory. We present moreover some performance evaluation tests that confirm the efficiency of RIN*maker*.

### Using RIN*maker* for data analysis of a molecular dynamics trajectory

One of the added values of mapping a 3D structure into a network stems from the various probes that can be used to identify emerging patterns that are not immediately visible from the original structure. We provide here an interesting example of this idea using RIN*maker*, thus showing the efficiency of the tool within this framework.

Consider Trp-cage (file 1L2Y.pdb in the Protein Databank [20]), a small protein made of 20 aminoacids. Since it is a fast folding protein (folding time in the nsec time domain), it has been thoroughly studied and its structure and properties are rather well understood [21, 22].

**Fig. 3:**
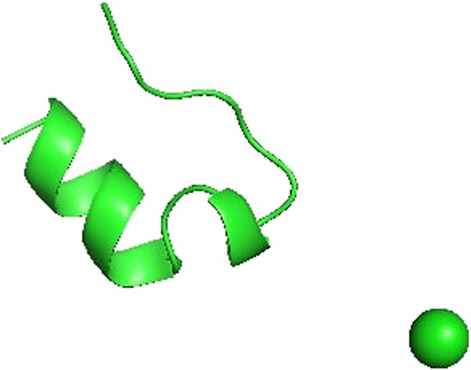
Native state of the Trp-Cage miniprotein with a $Cl^{-}$ as counter ion (Model 1 in the 1L2Y.pdb file)

The Trp-cage native 3D structure is depicted in Fig. 3 and contains a short $\alpha$-helix in residues 2–9, a 310-helix in residues 11–14, and a C-terminal polyproline II helix to pack against the central tryptophan (Trp-6) [21]. The latter residue appears to be involved in the formation/breaking of side-chain bonds during the various folding-unfolding processes, because of its preferential position in the core region [22]. Using molecular dynamics in explicit water TIP3P [23] with the CHARMM36 force-field [24], we first equilibrated the Trp-cage protein for 45 nsec in NPT ensemble with pressure equal to 1 atm kept constant with a Monte Carlo barostat [25]. Then a NVT production run of additional 45 nsec was performed at temperature 480K and at temperature 330 K. In all cases it was used a Langevin Integrator with timestep set to 2 psec. All simulations were performed using the OpenMM [26] python package.

From [21], it is known that with the use of the CHARMM36 force field, the ground-state (folded state) of the protein is well maintained at 330K, and that the melting point corresponding to denaturation of the protein, appears around 440K. Other studies show that the folded state is preserved exclusively by Trp at position 6 in the protein in the folded-state, while a denaturation involves a disruption of Trp’s noncovalent interactions with other nearby residues. Thus, the choice of temperatures at 330K and 480K were selected to emphasize these two different states of the protein in the free energy landscape.

During the production run, a number of reaction coordinates can be computed to highlight the behaviour of the protein at that specific temperature. In this case, we selected the radius of gyration that signals the onset of the folding of the protein, and the betweenness centrality computed on the RIN obtained via the use of RIN*maker*. The betweenness centrality is the ratio between the number of shortest paths passing through a specific node (i.e. a residue) and the number of total shortest paths in the network. The betweenness centrality is known to be an important indicator of the residue function of a protein upon a mutation of a given residue [27], and we show that this information is indeed captured by the RIN topology via our RIN*maker* tool.

For each snapshot of the trajectory (every 30ps), the radius of gyration and the RIN were computed. The latter was used to further compute the betweenness centrality using the Networkx [28] python package. Both these quantities were then accumulated in histograms that were then used for a free energy landscape analysis following standard prescriptions [21].

**Fig. 4:**
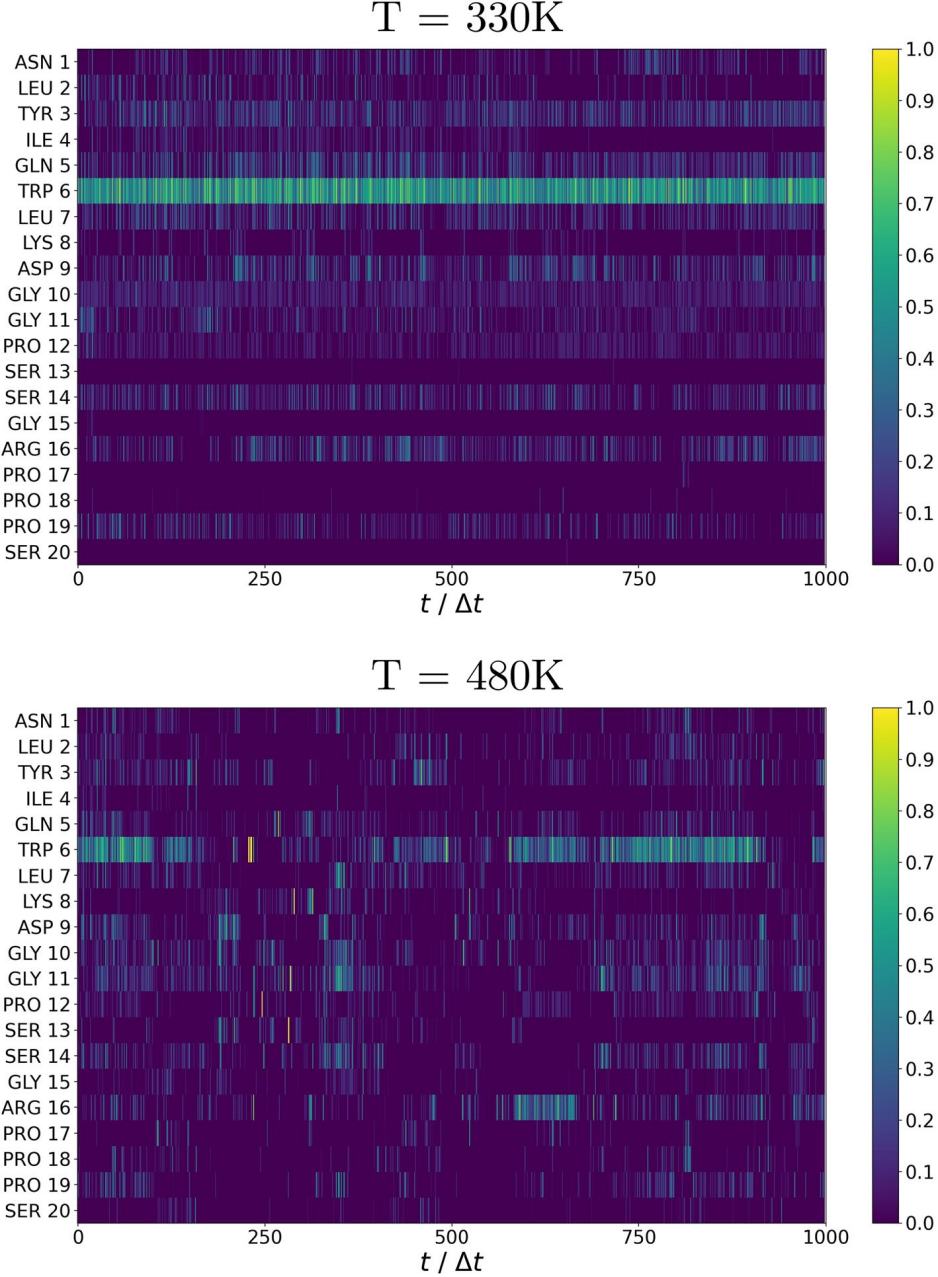
Betweenness centrality of the Trp-Cage residues as a function of the simulation time $t/\Delta t$ ($\Delta t=30$ psec) during the trajectory at temperatures above and below the folding temperature ($\approx 400K$). Top panel $T=330\,K$; Bottom panel $T=480\,K$

**Fig. 5:**
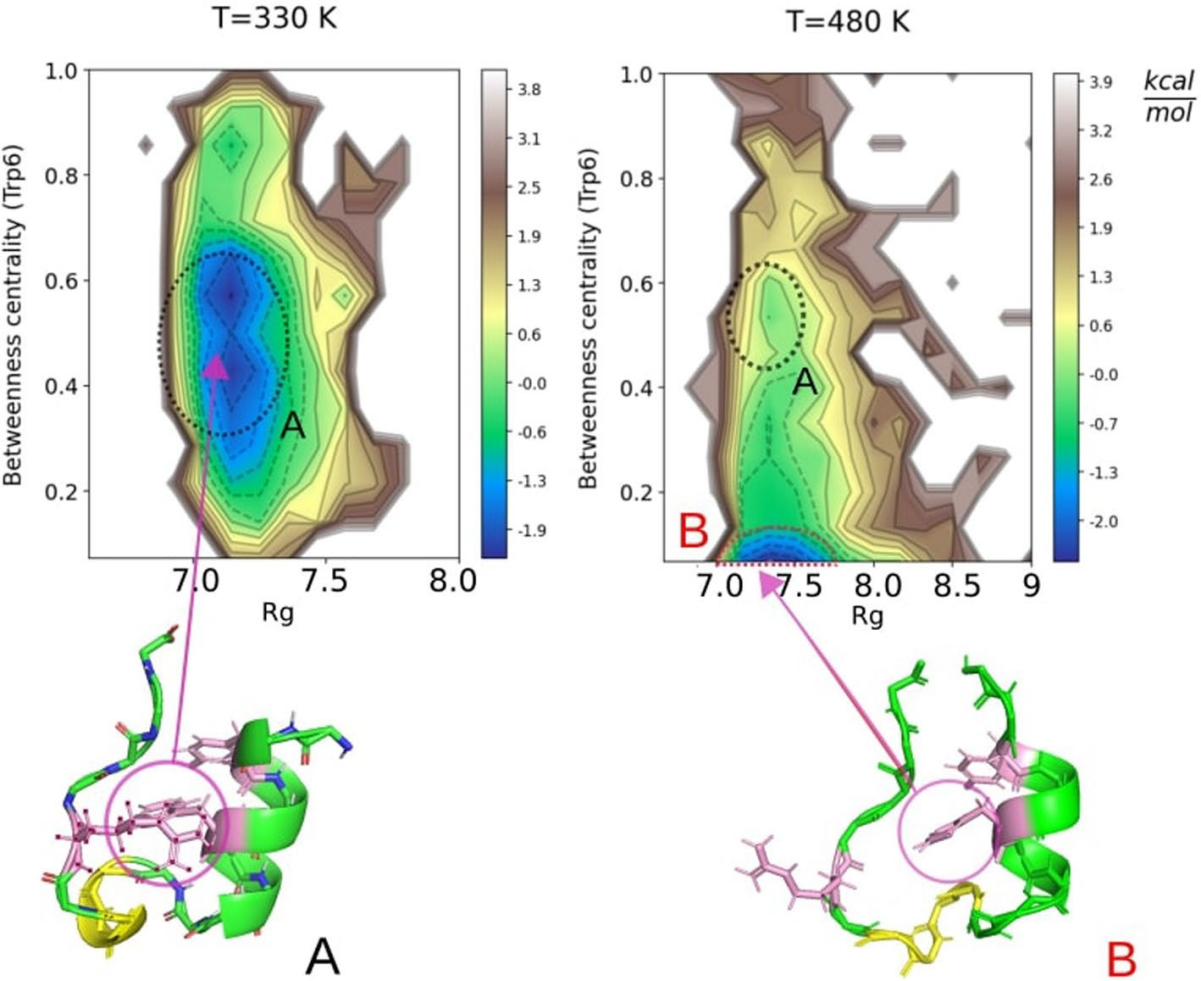
Free energy landscape of Trp-Cage as a function of the radius of gyration and of the betweenness centrality at temperatures (left) T = 330 K and (right) T = 480 K. Snapshots of the conformation corresponding to stable local minima are given in **A** for T = 330 K and in **B** for T = 480 K with highlighted the Gly11, Arg16 and Tpr6 residues involved in the transition between one minimum to the other. In **B** it is also possible to observe the denaturated $\alpha$-helix highlighted in yellow

Figure 4 displays the betweenness centrality of each of the 20 residues of the Trp-Cage as a function of the simulation time $t/\Delta t$ (units of $\Delta t =30$ psec) for two temperatures: (top panel) T = 330 K below the folding temperature ($T \approx 400\,K$) and (bottom panel) T = 480 K above it. The primary role played by Trp6 at T = 330 K can be clearly appreciated at a glance, and it agrees with past results [29] as further elaborated below. Above the folding temperature, precisely at T = 480 K, this prominence is lost at an early stage of the simulation and other residues, notably Arg16 and Gly11, begin to display their importance. This can be rationalized with the breaking and reforming of bonds as well as the stabilization of another local minimun. This process is captured by the free energy landscape calculations as illustrated in Fig. 5.

Figure 5 reports a contour plot of the free energy landscape as a function of the radius of gyration and the betweenness centrality at two different temperatures: $T=330\,K$ (left panel) and $T=480\,K$ (right panel). As the folding temperature is expected to be around 400K [30], the first temperature is below the folding temperature whereas the second is above.

In agreement with previous studies [31], we notice that at T = 330 K there is a well defined minimum (A) corresponding to the native state. This minimum becomes unstable at T = 480 K, above the folding temperature, due to a partial denaturation of one particular alpha-helix, thus driving the system toward another minimum (B). It should be emphasized that while this scenario is fully consistent with previous studies [31], specific details are not in view of the different force fields and different reaction coordinates employed. In this respect, the use of the betweenness centrality, and in turn of RIN*maker*, has proven very useful as it provides a much cleaner free energy landscape compared with previous one, thus highlighting the importance of RIN*maker* as a computational tool that can be embedded into a computational pipeline.

**Fig. 6:**
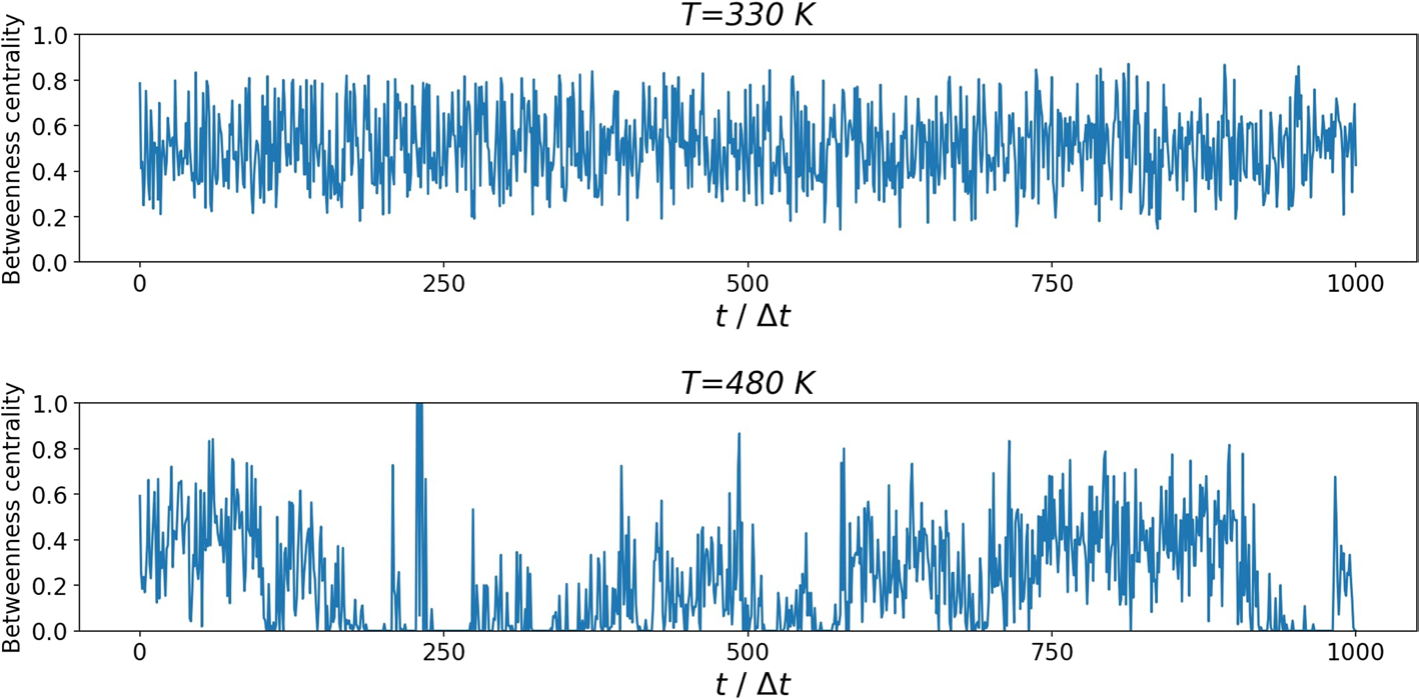
Betweenness centrality of residue Trp6 in the Trp-Cage trajectory obtained from RIN*maker*, at the two different temperatures (T = 330 K and T = 480 K)

The results reported in Fig. 4 have underpinned the fundamental role of Trp6 residue that is unveiled by the analysis of the betweenness centrality as obtained via RIN*maker*. Further insights can be obtained by plotting the actual value of betweenness centrality during the time evolution, as illustrated in Fig. 6. At 330 K the betweenness centrality remains stable, indicating an equlibrium localized in minimum A of Fig. 5. At 480K, the betweenness centrality displays a significant oscillatory behavior indicating a coexistence with another minimum (indicated as B in Fig. 5) throughout the entire trajectory. This can be ascribed to the salt-bridges and side-chain H-Bonds that this residue forms/breaks with the neighbour side-chains which shift the protein conformation between *A* and *B* [21]. The various spikes that can be seen in the process are probably due to the solvent noise and the transient interactions found by RIN*maker* when building the RIN.

### Performance evaluation

In this section we illustrate the performance tests that have been designed and performed for RIN*maker*. All tests were executed on a mid-end machine, namely a ThinkPad E590 laptop equipped with Intel i7-8565U CPU, 16GB of RAM and a 500GB SSD.

The first test aimed at evaluating the running time of the software for a wide set of proteins. We considered the TOP2018 [32] database and created a script to run RIN*maker* for all proteins stored in the database, which contains 13,678 entries (pdb files) corresponding to all single chains of the considered proteins. All the created RINs were obtained by using the default values for all input parameters. We measured that the average time to build the RIN for each entry was approximately 66 ms, for a total of 15 min for the entire database. We also computed the frequency per bond for the obtained RINs, which is reported in Table 1. The reported frequency values are in agreement with the general knowledge in the literature.

**Table 1:** Bonds frequency (%) calculated for the RINs of the TOP2018 database

Type of bond	Frequency (%)
VdW	57.5
H-Bond	25.5
Hydrophobic	16.2
Ionic	0.5
$\pi$–$\pi$ stacking	0.2
$\pi$-cation	0.1

The second test was a performance comparison between RIN*maker*, and the most similar tools in the literature, namely RING 3.0 [5] and PDBe-Arpeggio [10]. Note that, since RING 3.0 source code is unavailable, the test has been performed using the executables of the command-line version of the tool. For PDBe-Arpeggio we used the available Python scripts. While RIN*maker* and RING 3.0 are very similar in their general structure, PDBe-Arpeggio significantly departs from both on them on the count that it does not allow users to customize bond parameters (angles, distances, etc), and it heavily relies on external tools such as Open Babel [33].

The test aimed at evaluating the performance of the three tools for a small set of proteins available in the PDB databank and having an increasing number of amino acids. Table 2 shows the considered set of proteins and reports, for each of them, the *user CPU time* needed by all the three tools for creating their corresponding RIN. Each reported value has been averaged over ten runs values. The addition of hydrogen atoms is an optional operation in RIN*maker* and RING 3.0, while it is always included in PDBe-Arpeggio. Hence, for RIN*maker* and RING 3.0, the user CPU time is reported for both cases (i.e. with and without hydrogen addition). We remark that for each protein and run, RIN*maker* and RING 3.0 received the same input parameters. However, it is worth mentioning that, although the two tools operated at the same conditions, they do not perform exactly the same activities. In particular:

- RING 3.0 run the DSSP algorithm for secondary structure prediction [34], which is not included in RIN*maker*;
- Both tools report the H-bonds, VdW bonds, Ionic bonds, $\pi -\pi$ stacks and $\pi -$Cations. However, RIN*maker* reports also the hydrophobic bonds, which are not included in RING 3.0.

By contrast, in PDBe-Arpeggio everything is pre-defined thus lacking on any possibility of a customized use, as already remarked.

**Table 2:** Performance tests on an Intel i7-8565U CPU: RIN*maker* versus RING 3.0 versus PDBe-Arpeggio

PDB name	Num. of residues	CPU time (s) with hydrogen addition			CPU time (s) without hydrogen addition	
		RIN*maker*	RING 3.0	PDBe-Arpeggio	RIN*maker*	RING 3.0
1al1	12	0.0885	0.948	1.22	0.005	0.961
1l2y	20	0.0987	0.996	1.51	0.005	0.963
1bk8	50	0.0934	0.991	8.17	0.004	0.989
1eod	100	0.0975	1.031	6.64	0.009	0.994
1hhq	150	0.108	1.077	15.26	0.009	1.017
1ab5	250	0.115	1.118	63.25	0.015	1.036
1bge	318	0.120	1.209	131.50	0.022	1.119
1b1y	500	0.147	1.539	185.03	0.037	1.292
2vji	598	0.167	1.672	436.09	0.049	1.340
1miq	750	0.183	1.752	447.50	0.050	1.410
1a3w	1000	0.241	1.888	789.72	0.061	1.661
6j8j	1433	0.350	2.793	2245.17	0.084	2.210
1h1l	1994	0.604	3.417	7771.14	0.150	2.905
1i84	2382	0.506	3.594	$>8000.00$	0.143	2.923
3dwk	2444	0.619	4.067	$>8000.00$	0.185	3.416
3aae	2758	0.715	3.929	$>8000.00$	0.184	3.510
2qmi	3573	1.047	6.075	$>8000.00$	0.269	5.316
7fd2	4060	1.35	6.640	$>8000.00$	0.259	5.717

Looking at Table 2 it is clear that RIN*maker* outperforms PDBe-Arpeggio and RING 3.0 in all tests by at least one order of magnitude in most cases. It is also interesting to point out that the low user CPU times employed by RIN*maker* when dealing with small molecules imply that the program startup time, consisting of parsing the input and setting up data structures, is negligible compared to the processing time. This feature makes RIN*maker* ideal to be embedded into other tools, scripts or pipelines.

**Fig. 7:**
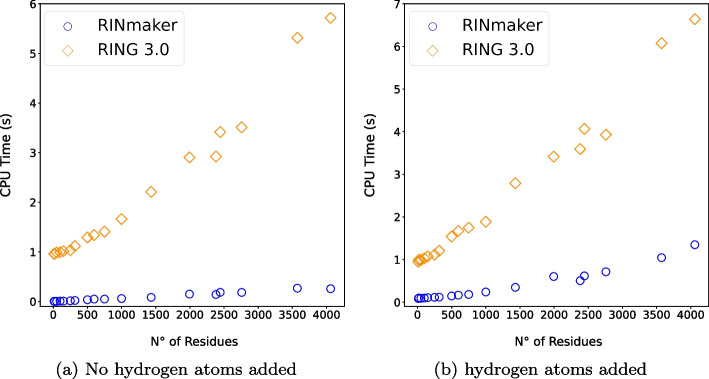
Performance tests: CPU time as a function of the number of residues. RIN*maker* (blue) vs. RING 3.0 (orange)

A different view of the comparison between RIN*maker* and RING 3.0 is shown in Fig. 7. It considers the user CPU time distribution w.r.t. the number of residues in the input files and shows the results of both tools in the two cases, namely with and without hydrogen addition. The results highlights even more the different performances of the two tools, RIN*maker* being the most efficient one. Note that the RIN*maker* CPU times grow very slowly w.r.t. the number of residues (it is a linear growth in case no hydrogen atoms are added), making it suitable for proteins of any size.

## Conclusion

RIN*maker* has been designed to be a flexible, reliable and fast tool to build and visualize RINs. Future developments of RIN*maker* include the extension of its web interface with additional visualization features as well as detailed analyses on the obtained RINs. In the current version, the web interface allows the 2D and 3D RIN visualization and offers some basic statistics. We would like to include the topological analysis of the obtained RIN and specific analyses associated to RINs determined on a MD trajectory, which could reveal interesting information on the simulated protein. Moreover, RIN*maker* is going to be linked to XRmol [35, 36], a web-based protein structure viewer that provides interactive 3D display of proteins and nucleic acids in different environments, including desktop computers and devices supporting Augmented Reality. The combination of the two tools, possible thanks to the web API available for RIN*maker*, will allow for depicting all non-covalent bonds calculated by RIN*maker* directly in the protein’s 3D structure visualized by XRmol.

The importance of having an efficient tool to construct RINs should not be underestimated. RINs provide a graph theoretical representation of a protein (or a protein domain) that captures the crucial complexity of the biomolecule at a glance and translates it into a set of quantitative indicators that are crucial to understand its function. A major challenge is to be able to achieve this dimensional reduction without loosing crucial information of its high-dimensional conformational space.

Efforts along these lines have recently been proposed [37] whose findings are complementary to ours thus providing further support on the impact that the tool proposed in this study may have in the near future.

### Supplementary Information

**Additional file 1.** This document contains detailed information about non-covalent bonds and test cases.

## Data Availability

Data analyzed in this paper are available at the PDB Data Bank [20] and at the TOP2018 database [32]. Both of them are public databases. **Availability and requirements**: Program name: RIN*maker*; Web UI: https://rinmaker.dais.unive.it/; Programming language: C++; Other requirements: none. License: The code is open source and available at github.com/RINmaker under the GNU Affero General Public License. Downloads: Binaries are available for multiple operating systems at github.com/RINmaker/RINmaker/releases. Contact: rinmaker@unive.it. Any restrictions to use by non-academics: none.
