# Supplementary material for: Rinmaker: a fast, versatile and reliable tool to determine residue interaction networks in proteins
Source: BMC Bioinformatics. 2023 Sep 11;24:336. doi: 10.1186/s12859-023-05466-y (PMC10496328; doi:10.1186/s12859-023-05466-y)
Supplement: Supplementary file 1 — Additional file 1. This document contains detailed information about non-covalent bonds and test cases. [file 12859_2023_5466_MOESM1_ESM.pdf]

Supplementary material to
RIN*maker*: a fast, versatile and reliable tool to determine residue interaction networks in proteins

– Non-covalent bonds and test cases –

Alvise Spanò[1], Lorenzo Fanton[1], Davide Pizzolato[1],
Jacopo Moi[2], Francesco Vinci[1], Alberto Pesce[1],
Cedrix J. Dongmo Foumthuim[2], Achille Giacometti[2,3] and Marta Simeoni[1,3,*]

[1] Department of Environmental Science, Computer Science and Statistics, University Ca' Foscari of Venice, 30172 - Venice, Italy.
[2] Department of Molecular Science and Nanosystems, University Ca' Foscari of Venice, 30172 - Venice, Italy.
[3] European Centre for Living Technology (ECLT), 30123 - Venice, Italy.
[*] corresponding author

1 Introduction

This document contributes to complete the presentation of RIN*maker*. Section 2 presents the details of the considered non-covalent bonds and explains the rules employed by the program to find them in a PDB or mmCIF file. Section 3 illustrates the test cases that have been designed and performed to ensure the program correctness.

2 Non-covalent interactions: general principles and main determination criteria

Non-covalent or non-bonded interactions can involve atoms in the same molecule (intramolecular non-bonded interactions) or atoms from different molecules (intermolecular non-bonded interactions). Although main biological assemblies such as proteins are primarily structured via covalent bonds forming the backbone skeleton, non-covalent counterpart appears to play a significant role in regulating essential biological functions. Hereafter, the mostly occurent type of nonconvalents interactions are described and the basic physicochemical laws underlining their nature and the mathematical backgrounds governing their determination are reviewed.

2.1 Ionic Interactions (Salt bridges)

Ionic interactions or salt bridges are defined as electrostatic interactions involving two molecular entities of opposite charges. In proteins and at near neutral pH, the following residues are charged and therefore potentially formed salt bridges : Asp(OD*), Glu(OE*) for negatively charged; Lys(NZ), Arg(NH), His(ND1) for positively charged. The ionic interaction is said to exist if the distance d between the centre of mass of the charged groups involved is less than 5 Å. From the mathematical point of view, ionic interactions are computed through Coulombic potentials as in Eq.1 below :

$$E_{Elec} = \frac{1}{4\pi\epsilon_0} \times \frac{q_1 q_2}{d} \tag{1}$$

where ϵ_0 is the vacuum permittivity, $d = r_{1,2}$ is the distance separation between the centre of mass of the groups 1 and 2 involved, q_1 and q_2 are the ionic charges. $\frac{1}{4\pi\epsilon_0} = 9 \times 10^9 Nm^2C^{-2}$.

The general overview of an ionic interaction is graphycally illustrated by Fig.S1.

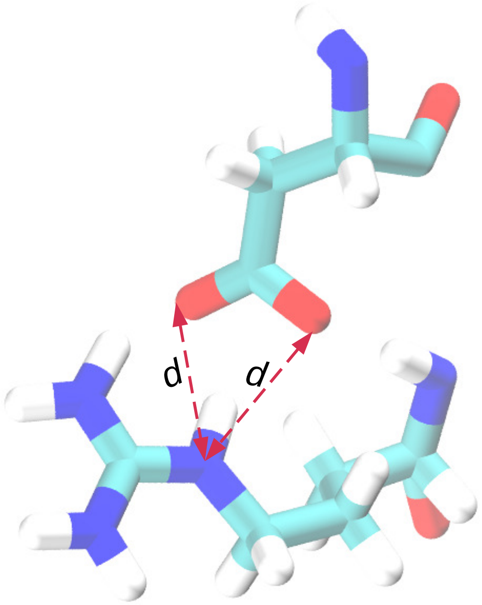

Figure S1: Illustration of an ionic interaction between two oppositely charge groups. d is the distance treshold defining the interaction.

The centre of mass of the charged centers can be estimated using the formulations schematized in Figs.S2 & Fig.S3 below.

POSITIVE AMINO ACIDS

CM=[9]NZ

LYS (Lysine)

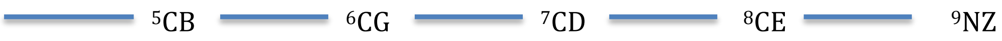

ARG (Arginine)

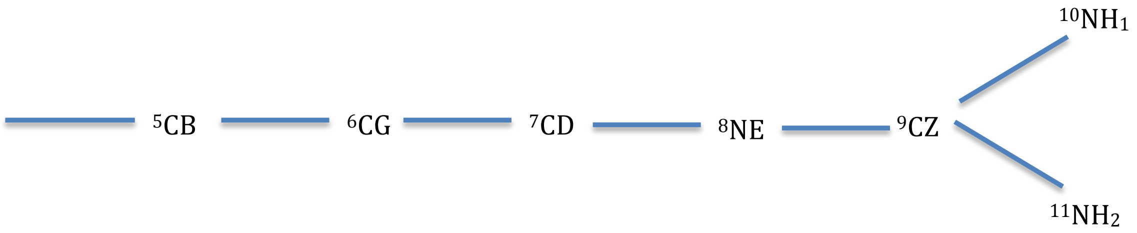

$$x_{CM} = \frac{M_{{}^{8}NE}x_{{}^{8}NE} + M_{{}^{9}CZ}x_{{}^{9}CZ} + M_{{}^{10}NH_1}x_{{}^{10}NH_1} + M_{{}^{11}NH_2}x_{{}^{11}NH_2}}{M_{{}^{8}NE} + M_{{}^{9}CZ} + M_{{}^{10}NH_1} + M_{{}^{11}NH_2}} \qquad y_{CM} = \frac{M_{{}^{8}NE}y_{{}^{8}NE} + M_{{}^{9}CZ}y_{{}^{9}CZ} + M_{{}^{10}NH_1}y_{{}^{10}NH_1} + M_{{}^{11}NH_2}y_{{}^{11}NH_2}}{M_{{}^{8}NE} + M_{{}^{9}CZ} + M_{{}^{10}NH_1} + M_{{}^{11}NH_2}}$$

$$z_{CM} = \frac{M_{{}^{8}NE}z_{{}^{8}NE} + M_{{}^{9}CZ}z_{{}^{9}CZ} + M_{{}^{10}NH_1}z_{{}^{10}NH_1} + M_{{}^{11}NH_2}z_{{}^{11}NH_2}}{M_{{}^{8}NE} + M_{{}^{9}CZ} + M_{{}^{10}NH_1} + M_{{}^{11}NH_2}}$$

HIS (Histidine)

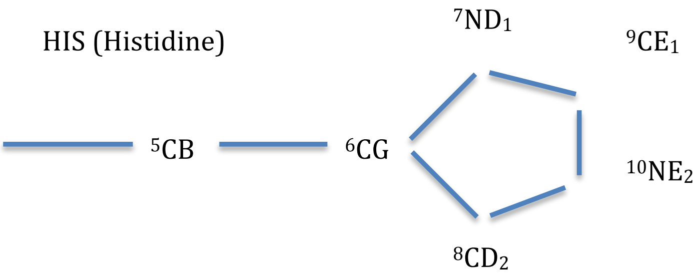

$$x_{CM} = \frac{M_{{}^{6}CG}x_{{}^{6}CG} + M_{{}^{7}ND1}x_{{}^{7}ND_1} + M_{{}^{8}CD_2}x_{{}^{8}CD_2} + M_{{}^{9}CE_1}x_{{}^{9}CE_1} + M_{{}^{10}NE_2}x_{{}^{10}NE_2}}{M_{{}^{6}CG} + M_{{}^{7}ND1} + M_{{}^{8}CD_2} + M_{{}^{9}CE_1} + M_{{}^{10}NE_2}}$$

$$y_{CM} = \frac{M_{{}^{6}CG}y_{{}^{6}CG} + M_{{}^{7}ND1}y_{{}^{7}ND_1} + M_{{}^{8}CD_2}y_{{}^{8}CD_2} + M_{{}^{9}CE_1}y_{{}^{9}CE_1} + M_{{}^{10}NE_2}y_{{}^{10}NE_2}}{M_{{}^{6}CG} + M_{{}^{7}ND1} + M_{{}^{8}CD_2} + M_{{}^{9}CE_1} + M_{{}^{10}NE_2}}$$

$$z_{CM} = \frac{M_{{}^{6}CG}z_{{}^{6}CG} + M_{{}^{7}ND1}z_{{}^{7}ND_1} + M_{{}^{8}CD_2}z_{{}^{8}CD_2} + M_{{}^{9}CE_1}z_{{}^{9}CE_1} + M_{{}^{10}NE_2}z_{{}^{10}NE_2}}{M_{{}^{6}CG} + M_{{}^{7}ND1} + M_{{}^{8}CD_2} + M_{{}^{9}CE_1} + M_{{}^{10}NE_2}}$$

Figure S2: Positively charged aminoacids along with an approximate formulation of their center of mass, where M_i are the molecular masses of individual atoms involved.

NEGATIVE AMINO ACIDS

GLU (Glutamic Acid)

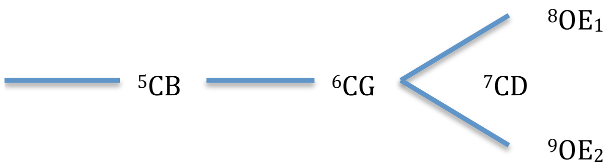

$$x_{CM} = \frac{M_{{}^{7}CD}x_{{}^{7}CD} + M_{{}^{8}OE_1}x_{{}^{8}OE_1} + M_{{}^{9}OE_2}x_{{}^{9}OE_2}}{M_{{}^{7}CD} + M_{{}^{8}OE_1} + M_{{}^{9}OE_2}}$$

$$y_{CM} = \frac{M_{{}^{7}CD}y_{{}^{7}CD} + M_{{}^{8}OE_1}y_{{}^{8}OE_1} + M_{{}^{9}OE_2}y_{{}^{9}OE_2}}{M_{{}^{7}CD} + M_{{}^{8}OE_1} + M_{{}^{9}OE_2}}$$

$$z_{CM} = \frac{M_{{}^{7}CD}z_{{}^{7}CD} + M_{{}^{8}OE_1}z_{{}^{8}OE_1} + M_{{}^{9}OE_2}z_{{}^{9}OE_2}}{M_{{}^{7}CD} + M_{{}^{8}OE_1} + M_{{}^{9}OE_2}}$$

ASP (Aspartic Acid)

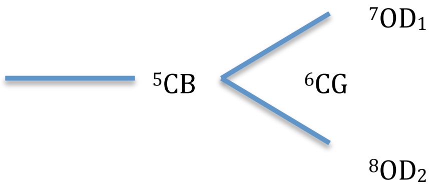

$$x_{CM} = \frac{M_{{}^{6}CG}x_{{}^{6}CG} + M_{{}^{7}OD_1}x_{{}^{7}OD_1} + M_{{}^{8}OD_2}x_{{}^{8}OD_2}}{M_{{}^{6}CG} + M_{{}^{7}OD_1} + M_{{}^{8}OD_2}}$$

$$y_{CM} = \frac{M_{{}^{6}CG}y_{{}^{6}CG} + M_{{}^{7}OD_1}y_{{}^{7}OD_1} + M_{{}^{8}OD_2}y_{{}^{8}OD_2}}{M_{{}^{6}CG} + M_{{}^{7}OD_1} + M_{{}^{8}OD_2}}$$

$$z_{CM} = \frac{M_{{}^{6}CG}z_{{}^{6}CG} + M_{{}^{7}OD_1}z_{{}^{7}OD_1} + M_{{}^{8}OD_2}z_{{}^{8}OD_2}}{M_{{}^{6}CG} + M_{{}^{7}OD_1} + M_{{}^{8}OD_2}}$$

Figure S3: Negatively charged aminoacids along with an approximate formulation of their center of mass, where M_i are the molecular masses of individual atoms involved.

2.2 Hydrogen bonds

Hydrogen bonds are non-covalent interactions established between a hydrogen atom (H) covalently bond to a more electronegative or polar atom called donor (D) and another electronegative atom usually bearing a lone pair of electrons, i.e. willing to accept the hydrogen atom, called acceptor (A). Typical

electronegative atoms include O, N, S and F. A schematic illustration of a hydrogen bond is shown in Fig.S4.

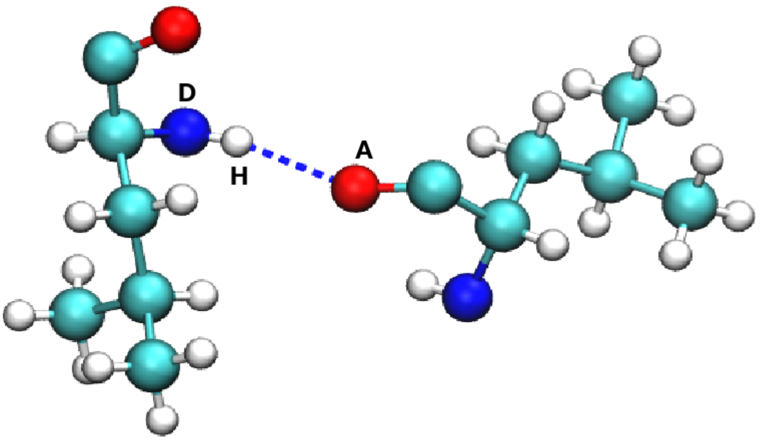

Figure S4: Schematic representation of hydrogen interaction shown by blue intercepted lines between two chemical moieties.

Hydrogen bonds are determined through geometrical criteria and the following conditions on distance and angle should be fulfilled [10] :

1. The distance d between Acceptor (A) and Donor (D) should be less than or equal to 3.5 Å , and in less restrained cases extended to 5.5 Å, i.e. $0 < d \leq 3.5(5.5)$ Å

2. The Hydrogen-Donor-Acceptor $\widehat{HDA}$ angle, α, should be less than or equal to 63°, i.e. $\alpha \leq 63°$

In proteins, as hydrogen bonds can be either intra-molecular (atoms within the same molecule) or inter-molecular (atoms from different molecules), we can distinguish between several types of hydrogen bonds :

1. **Main chain - main chain hydrogen bonds** : when the hydrogen donor is the main chain backbone NH and the acceptor is the main chain backbone carbonyl (C=O)

2. **Main chain - side chain hydrogen bonds** : when the hydrogen donor is the main chain backbone NH and the acceptor arises from the side chains R, or the hydrogen donor comes from the side chains R and the acceptor is the backbone main chain carbonyl C=O.

3. **Side chain - side chain hydrogen bonds** : when both hydrogen donor and acceptor come from the side chains R.

Typical side-chain hydrogen donors and acceptors are summarized in table S1 below. However, it should be remind that nine amino acids (alanine, cysteine, glycine, isoleucine, leucine, methionine, phenylalanine, proline, and valine) do not have hydrogen donor or acceptor atoms in their side-chains and thus cannot be involved in side chain - side chain hydrogen bond types. In general, the shorter the D-A distance, the stronger the hydrogen bond interaction.

Table S1: Hydrogen donor and acceptor atoms in amino acid side chains.

Amino acids	Hydrogen donor atoms$^{(1)}$	Hydrogen acceptor atoms$^{(2)}$
Arginine (Arg, R)	NE, NH1 (2), NH2 (2)	
Asparagine (Asn, N)	ND2 (2)	OD1 (2)
Aspartic acid (Asp, D)		OD1 (2), OD2 (2)
Glutamine (Gln, Q)	NE2 (2)	OE1 (2)
Glutamic acid (Glu, E)		OE1 (2), OE2 (2)
Histidine (His, H)	ND1, NE2	ND1, NE2
Lysine (Lys, K)	NZ (3)	
Serine (Ser, S)	OG	OG (2)
Threonine (Thr, T)	OG1	OG1 (2)
Tryptophan (Trp, W)	NE1	
Tyrosine (Tyr, Y)	OH	OH

$^{(1)}$ *: In brackets, number of hydrogens that a donor atom can donate, if more than one*
$^{(2)}$ *: In brackets, number of hydrogen bonds that an acceptor atom can accept, if more than one*

To model the hydrogen bond interaction some force fields replace the Lennard-Jones 12-6 term between hydrogen-bonding atoms by an explicit hydrogen-bonding term, likely described by a 10-12 Lenard Jones potential. So, the strength of a h-bond interaction can be estimated as follows [17, 13]:

$$E_{HB} = 4\epsilon_{ij}\left[\left(\frac{\sigma_{ij}}{r_{ij}}\right)^{12} - \left(\frac{\sigma_{ij}}{r_{ij}}\right)^{10}\right] \tag{2}$$

where ϵ_{ij} is the van der Waals well depth and σ_{ij} is the distance at which $E_{HB} = 0$. The corresponding values for these latter parameters are reported in tableS2 below [17]:

Table S2: Nonbonded 12/10 hbonds parameters.

equilibrium distance/Å	well depth/kcalmol^{-1}	hbond type
1.99	-3.00	N—H$\cdots$N for any charge model
1.89	-3.50	N—H$\cdots$O for any charge model
1.89	-4.00	O—H$\cdots$N for any charge model
1.79	-4.25	O—H$\cdots$O for any charge model
1.99	-4.50	+N—H$\cdots$N for zero-charge model only
1.89	-5.25	+N—H$\cdots$O for zero-charge model only
1.89	-5.25	N—H$\cdots$O— for zero-charge model only
1.89	-7.00	+N—H$\cdots$O— for zero-charge model only
1.79	-6.375	O—H$\cdots$O— for zero-charge model only

2.3 $\pi - \pi$ Stacking

π-stacking interactions are ubiquitous inter-molecular interactions between conjugated molecules as graphically exemplified in Fig. S5. Also termed $\pi - \pi$ interactions, they are associated with the interactions between the π-orbitals of a molecular system, for e.g. benzene rings. Typical aromatic stacking arrangements [14], as shown in Fig. S6, include:

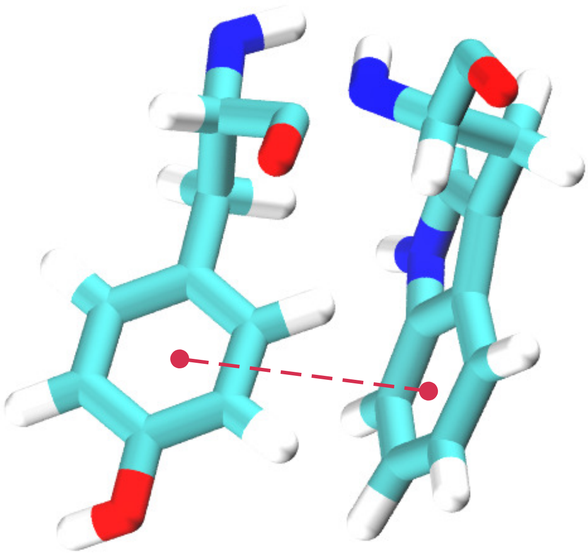

Figure S5: General overview of $\pi - \pi$ stacking.

1. Offset stacked (parallel displaced) ;
2. Edge-to-face (T-shaped);
3. Face-to-face (sandwich).

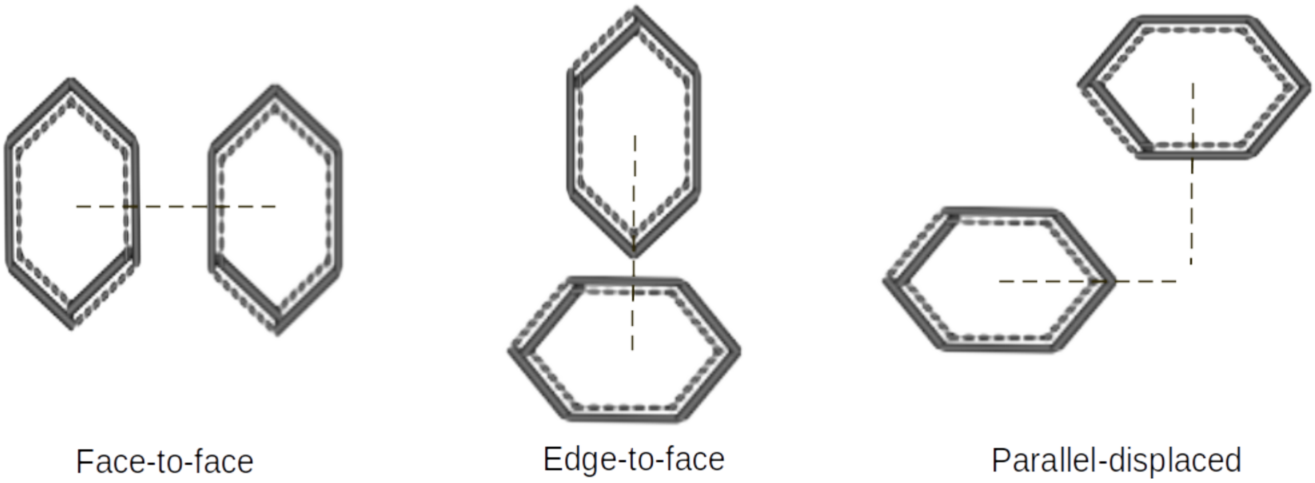

Figure S6: Types of $\pi - \pi$ stacking interactions between aromatic rings.

Fundamentally, the following rules should be taken into account for the existence of a $\pi - \pi$ non-covalent interaction, as schematically illustrated by Fig.S7 [3, 11, 8]:

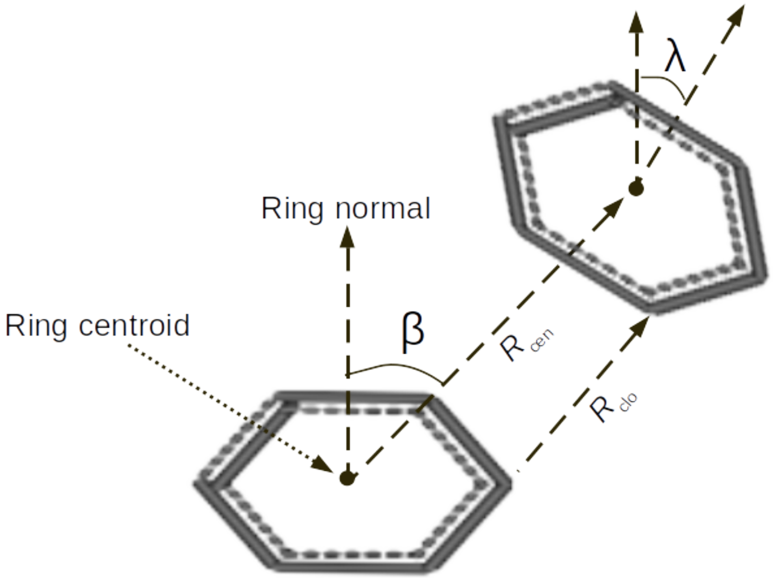

Figure S7: Geometric parameters describing a $\pi - \pi$ interaction between two rings.

1. The distance R_{cen} between the centroid of each pair of π rings should fall within 8Å, i.e. $R_{cen} \leq 8$ Å;
2. At least one atom from each ring (R_{clo}) should be within 4.5 Å, i.e. $R_{clo} \leq 4.5$ Å;
3. The angle β between the normal of one or both rings and the centroid-centroid vector must fall between 0 and $\pm60°$;
4. The angle λ between the normal of each ring must fall between 0 and $\pm30°$.

Previous statistical analysis from the PDB database [19] and quantum chemical calculations [15] reported that T-shaped and offset stacked conformations are energetically favoured over sandwich (face-to-face) one due their smaller electron repulsions, see Fig.S8.

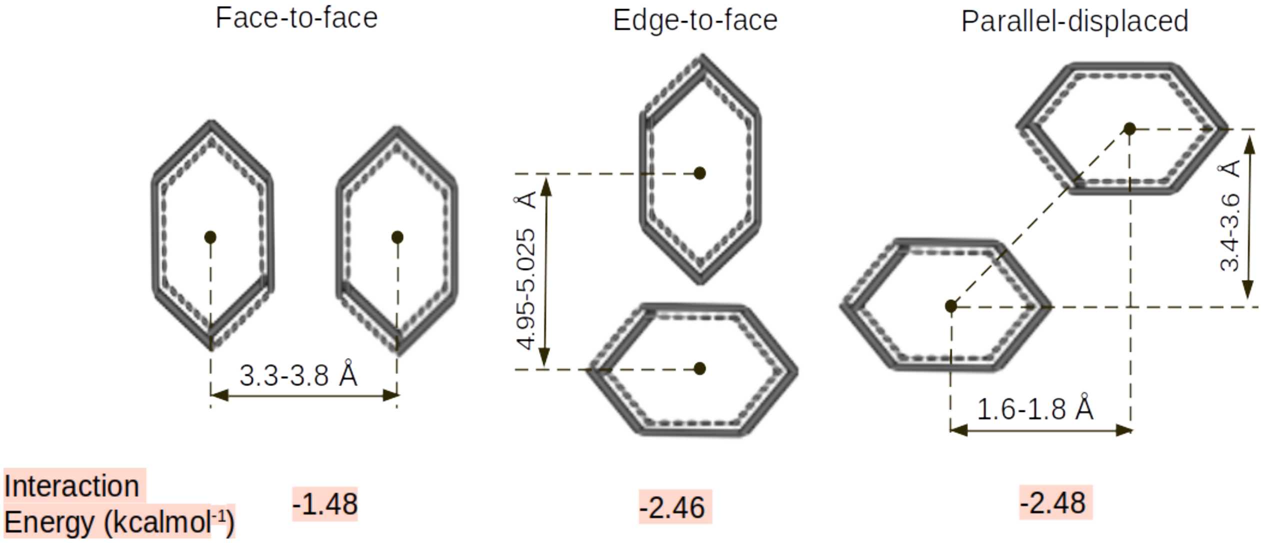

Figure S8: Energetic views of the representatives $\pi - \pi$ interaction.

One can use the angle between the two ring's normal γ to distinguish among the above π-π geometries as defined below [19] (Fig.S9) :

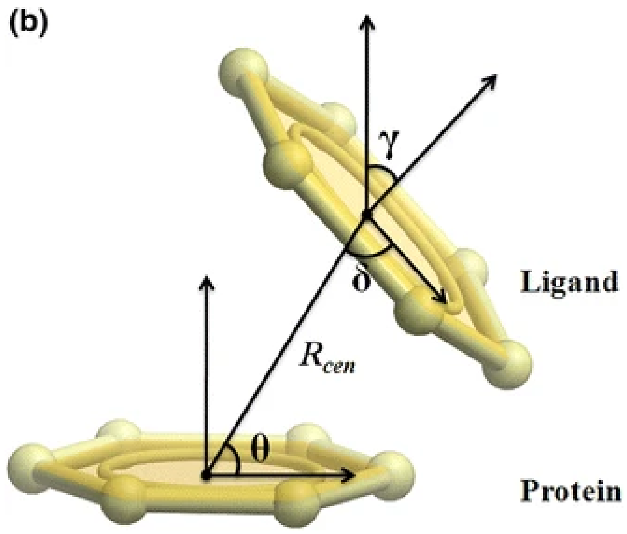

Figure S9: Spherical polar pair orientational coordinates in the case of a protein-ligand complex. R_{cen}: centroid-centroid distance; θ: acute angle between the centroid–centroid vector and the aromatic ring plane of protein; δ: acute angle between the centroid–centroid vector and the aromatic ring plane of ligand; γ: acute angle between the normal of the two rings planes.

1. Offset stacked (parallel displaced) : $\gamma < 30°$ and θ (or δ) $> 80°$
2. Edge-to-face (T-shaped) : $\gamma > 50°$
3. Face-to-face (sandwich) : $\gamma < 30°$

The estimation of pi-stacking energetic from the molecular mechanics perspectives is very tricky due the involvement of electronic clouds of interacting aryl rings. However, by fitting the data obtained from previous *ab initio* quantum mechanics calculations [15] we can fit the orientation dependence of the total interaction energy by the following empirical functional equation :

$$E_{\pi-\pi} = a + b\theta + c\theta\cos\left(\frac{1}{\theta+10}\right) \tag{3}$$

where θ is acute angle between the centroid–centroid vector and the aromatic ring plane (see Fig.S9) and the fitting coefficients a, b and c are -0.5274, 25.6290, and -25.653, respectively. The previous functional leads to correlation coefficient of 0.99749 with the plot reported in Fig.S10 below:

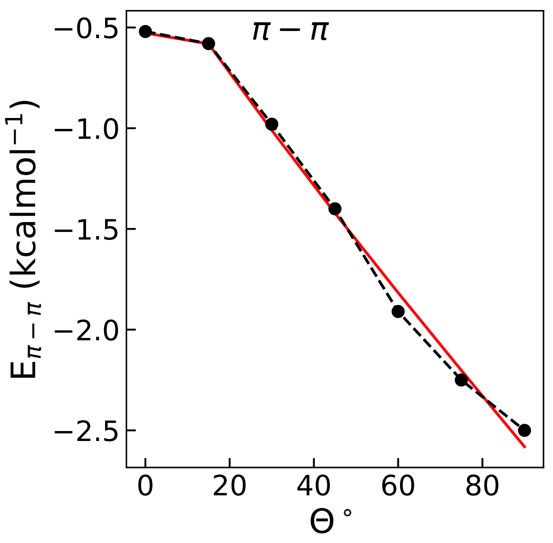

Figure S10: Orientation dependence of the total $\pi - \pi$ stacking interaction. The black represents the data taken from [15] and the red one the corresponding fitted curve. Θ is the angle between the centroid–centroid vector and one aromatic ring plane.

2.4 π-cation

A π-cation interaction is illustrated in Fig.S11.

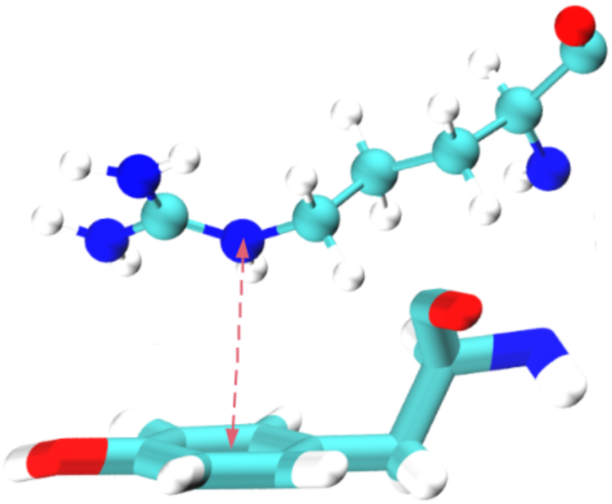

Figure S11: A molecular view of a π-cation interaction.

The rules governing the existence of this interaction are summarized hereafter and schematized in Fig. S12 [3] :

1. The distance R between each cation and the centroid of each π-ring should fall within 7 Å.
2. The angle θ between the cation-centroid vector and the ring plane should be more than the 45°. It should be pointed out that α is the dihedral angle between two extended planes.

The nature of the π-cation restricts the search among the following group of amino acid residues : LYS (NZ), ARG (NH), HIS (ND1) for potential cation-centre & PHE, TRP, TYR for potential π-ring providers. In the case of TRP residue, only the 6-membered ring is π-delocalized and thus can be involved in the cation-π interaction.

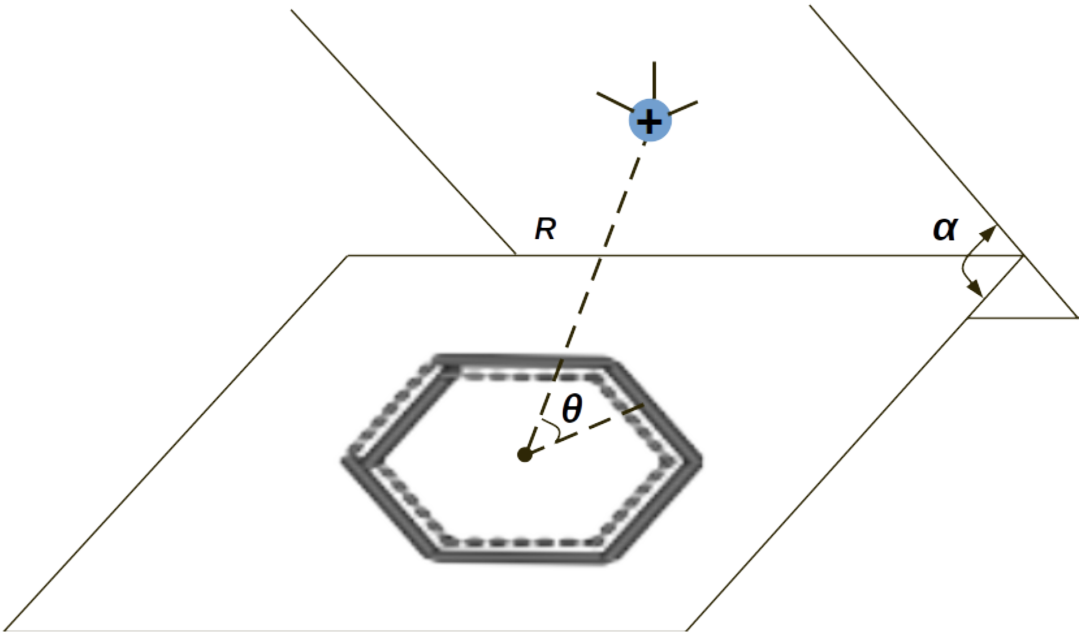

Figure S12: Schematic representation of a π-cation interaction.

The strength of the cation$-\pi$ interaction was recently modelled as $1/r^4$ in OPLS/2020 force field by adding to the nonbonded term a potential energy between site(s) on the cation i and aromatic atoms j, which have associated parameters κ and α to control the strength of the interaction, as in Eq.S12 [16]

$$E_{picat} = -\frac{\kappa_i \alpha_j}{r_{ij}^4} \tag{4}$$

The optimized values of κ and α are reported in table.S3 below [16]:

Table S3: Optimized κ and α parameters for OPLS/2020 force field.

type	atom	κ	type	atom	α
Li	Li^+	0.45	CA	benzene, aromatic C	190
Na	Na^+	0.70	CW	C2 in furan/thiophene	210
K	K^+	0.95	CS	C3 in furan/thiophene	210
Rb	Rb^+	0.70	NA	N in pyrrole/indole	150
Cs	Cs^+	0.75	NC	N in azine	100
N3	ammonium N	1.00	OA	O in furan	150
N2	guanidinium N	0.25	SA	S in thiophene	50

2.5 Van der Waals interaction

van der Waals (vdW) attraction is a distance-related interaction between molecular moieties, as shown in Fig. S13. It does not require any chemical electronic bonds unlike ionic/covalent bond and thus, is relatively weak and more susceptible to disturbance. vdW forces are simply accessed by considering the distance separation between the molecular surfaces of the interacting groups. So, vdW interaction exists if the distance d between the molecular surfaces of the molecules involved is less than 0.5 Å, or 0.8 Å in relaxed cases.

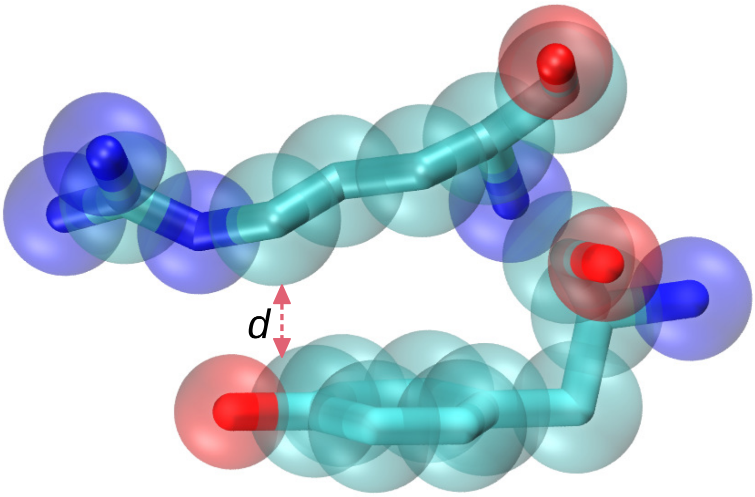

Figure S13: Schematic representation of van der Waals interaction. The molecular surfaces of interacting molecules are shown in balls representing their van der Waals spheres.

The van der Waals interaction is often computed as the Lenard-Jones 12-6 potentials as in Eq.5 :

$$E_{vdw} = 4\epsilon_{ij}\left[\left(\frac{\sigma_{ij}}{r_{ij}}\right)^{12} - \left(\frac{\sigma_{ij}}{r_{ij}}\right)^{6}\right] \tag{5}$$

in which ϵ_{ij} is the van der Waals well depth and σ_{ij} is the distance at which $E_{vdw} = 0$.

As shown in Eq.5, the van der Waals forces are divided into two contributions. The attractive component, which comes from the dispersive effects and has the basis in the dominant term of Drude (r^{-6}), and the repulsive component, which emerges from the compliance of the exclusion principle of Pauli when the electronic clouds of two atoms interpenetrate, although theoretical arguments do not exist for r^{-12} term, since the quantum mechanics suggest an exponential law.

The values of σ_{ij} and ϵ_{ij} are computed using the Lorentz-Berthelot combining rules by applying a geometrical average in the case of the OPLS force field [9] as given below in Eq.6 :

$$\begin{aligned} \sigma_{ij} &= (\sigma_{ii}\sigma_{jj})^{\frac{1}{2}} \\ \epsilon_{ij} &= (\epsilon_{ii}\epsilon_{jj})^{\frac{1}{2}} \end{aligned} \tag{6}$$

The atom types defined in OPLS force field as well as and their corresponding charges, σ_{ii}, and ϵ_{ii} values can be respectively retrieved from tables II, III, and IV of the original publication [9].

2.6 Hydrophobic-hydrophobic contacts

Hydrophobic interactions play a major role in the protein stability and are consistently accepted as a main driven force of protein folding. The constituent protein's amino acids are often referred as polar or hydrophobic based on the chemical nature of their side-chains. In the literature, several hydrophobic scales can be used with more or less overlapping ranking. However, the recent scale provided by some of the authors of the present work was used here [4]. According to the latter, the following residues were considered in counting hydrophobic contacts : ILE, LEU, VAL, MET, PHE, ALA, TRP, CYS, GLY, arranged in decreasing order of hydrophobicity. We further consider GLY in the current work as a hydrophobic amino acid residue, albeit it was not investigated previously [4].

The hydrophobic-hydrophobic contacts can be determined by computing the separation distance between the C_α atoms of hydrophobic residues. Indeed, C_α is often believed to represent a good approximation of the centre of mass of each naturally occurring amino acid [12]. Nevertheless, nearest neighbors C_α atoms are excluded from the counts and only inter-C_α atoms separated by at least one bond, i.e. between atom i and $i+3$ and beyond are considered. Therefore, hydrophobic-hydrophobic interactions are counted for all hydrophobic residues whose inter-C_α separation distances d are within 7.5Å [2, 18, 1].

To summarize :

1. identify all hydrophobic residues : ILE, LEU, VAL, MET, PHE, ALA, TRP, GLY;
2. compute the separation distance d between C_α atoms separated by at least 1 bond i.e. between atoms i and $i+3$, and beyond;
3. if $d \leq 7.5$ Å, then count the hydrophobic-hydrophobic interaction

The estimation of the strength of hydrophobic contacts is not straightforward especially in a solvent, so the expression used hereafter should be taken with due care. However, in vacuum, the interacting potential between 2 non polar solutes can be estimated based on London dispersion energy given in Eq.7 as follows [6, 7]:

$$E_{HH} = -\frac{3}{4}\frac{h\nu_n\alpha^2}{(4\pi\epsilon_0)^2 d^6} \tag{7}$$

where h is the Plank's constant ($6.626 \times 10^{-34} Js$), $\nu_n = (2\pi kT/h)n \approx 4 \times 10^{13} n\ s^{-1}$ at (300 K) is the electronic absorption (ionization) frequency, n is the refractive index of the medium equals to 1 in vacuum and 1.333 in water, α is the electric polarizability ($C^2 m^2 J^{-1}$), ϵ_0 is the dielectric permittivity of free space ($8.854 \times 10^{-12} C^2 J^{-1} m^{-1}$), and d is the separation distance between interacting atoms/molecules (m).

The table S4 reports on the experimental and computed values of the electronic polarizabilities for each hydrophobic residue considered in this work [5].

Table S4: Experimental and computed values of the electronic polarizabilities α of hydrophobic residues considered in this work in a.u.

Amino acid	experimental	computed$^{(1)}$
ILE	95.2	92
LEU	94.5	92
VAL	81.5	81
MET	102.1	101
PHE	122.9	123
ALA	55.9	58
TRP	157.8	150
CYS	–	77
GLY	44.3	46

$^{(1)}$ *: MP2/d-aug-cc-pVDZ*MP2/d-aug-cc-pVDZ

To calculate Eq. 7 RIN*maker* uses the approximated version of ν_n with $n = 1$. Moreover, it uses the experimental values for α (when possible) and computes α^2 as the multiplication of the electronic polarizabilities of the two involved hydrophobic residues.

2.7 Summary of noncovalent bonds

Table S5 reports the strength energies and the nature of noncovalent interactions. The interactions discussed in this work are highlighted in LightCyan.

Table S5: Table summarizing the strength energies and the nature of noncovalent interactions. The interactions discussed in this work are highlighted in LightCyan.

Noncovalent interactions	Bonding Energy ($kcalmol^{-1}$)	Nature of the Interaction
Ion-Ion	25 – 85	Electrostatic
Ion–Dipole	10 – 50	Electrostatic and Induction
Dipole–Dipole	1 – 10	Electrostatic and Induction
Hydrogen Bond	1 – 30	Electrostatic (dipole-dipole)
C–H. . . π	1	Weak Hydrogen Bond
Cation-π	1-20	Electrostatic and Induction
$\pi - \pi$ Stacking	1-10	Weak Electrostatic and Dispersion
Anion-π	5-10	Electrostatic and Induction
Lone pair-π	1-5	Electrostatic
Halogen Bond	1-45	Weak Electrostatic
Hydrophobic Effect	< 1	Thermodynamic
van der Waals Forces	< 1	Dispersion

3 Test cases

In this section we illustrate the tests that have been developed to check the correctness of RIN*maker*. We begin by showing the molecules designed for testing the various non-covalent bonds. We use tables, one for each bond type, to report the relevant information about such molecules, that is, the name of the PDB file containing the molecule, its picture and a brief description. In particular, Table S6 illustrates the molecules employed to test the ionic bonds, Table S7 illustrates the H-bond molecules, Table S8 shows the molecules designed for π-π stacking bonds, Table S9 depicts the molecules designed for VDW bonds, Table S10 shows the molecules for the $\pi-$cation bonds and finally Table S11 illustrates the molecules used for hydrophobic bonds.

File name (pdb)	Image	Description
IonIon1	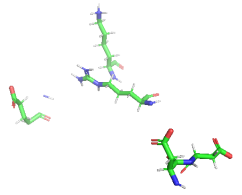	Test on an ensemble of 4 residues
IonIon2	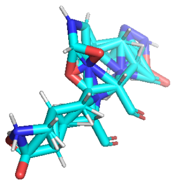	Two residues fulfilling ion-ion default bond rules
IonIon3	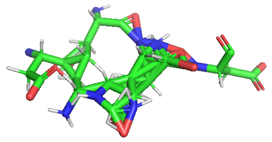	Three residues fulfilling ion-ion default bond rules
IonIon4	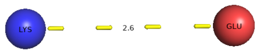	Two ionic groups (NZ, OE1) fulfilling default ion-ion bond rules
IonIon5	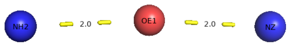	Three ionic groups (NH2,OE,NZ) fulfilling default ion-ion bond rules
IonIon6	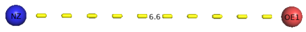	Two ionic groups (NZ,OE1) not fulfilling default ion-ion rules
IonIon7	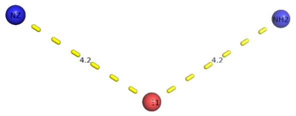	Three ionic groups (NH2,OE,NZ) not fulfillig default ion-ion bond rules
IonIon8	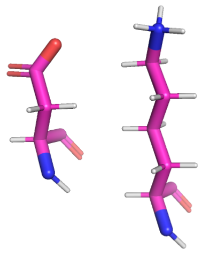	Two residues (LYS-ASP) fulfilling default ion-ion bond rules
IonIon9	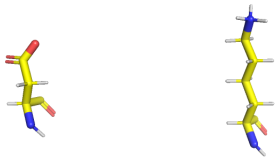	Two residues (LYS-ASP) not fulfilling ion-ion bond rules

Table S6: Molecules specifically designed for Ionic bonds tests.

File name (pdb)	Image	Description
HBond1	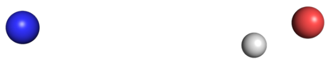	One donor group and one acceptor group potentially H-Bond bonded not fulfilling *d* and angle parameter
HBond2	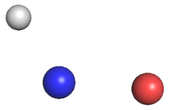	Two donor and acceptor groups potentially H-Bond not fulfilling the angle parameter
HBond3	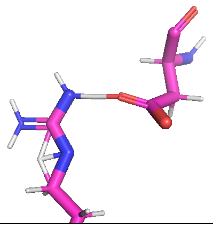	Two H-Bonded residues fulfilling the default rules
HBond4	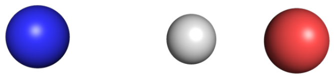	Two H-Bonded residues fulfilling default H-Bond rules
HBond5	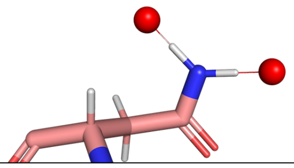	One residue (ASN) with two donor groups (OD)
HBond6	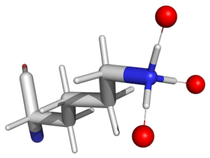	One residue (LYS) and a three donor groups (OH) fulfilling the default H-Bond rules
HBond7	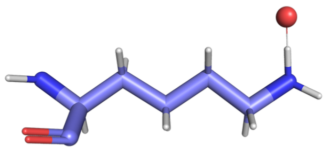	One residue (LYS) and one donor group (OD) fulfilling H-Bond rules
HBond8	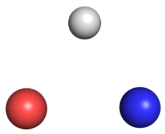	Two H-Bonded residues fulfilling the default rules

Table S7: Molecules specifically designed for H-bonds tests.

File name (pdb)	Image	Description
Pipi1	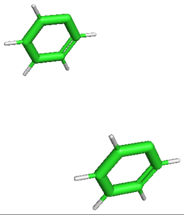	Two aromatic rings (Benzene) not fulfilling standard rules for π-π stacking bond
Pipi2	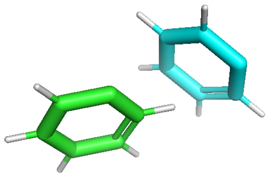	Two aromatic rings (Benzene) not fulfilling standard rules for π-π stacking bond
Pipi3	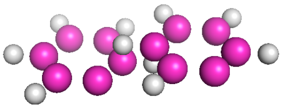	Two aromatic rings (Benzene) not fulfilling standard rules for π-π stacking bond
Pipi4	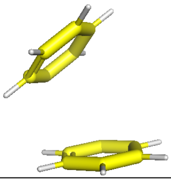	Two aromatic rings (Benzene) not fulfilling standard rules for π-π stacking bond
Pipi5	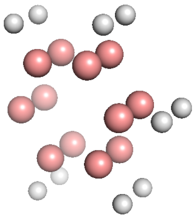	π-π stacking fulfilling rules
Pipi6	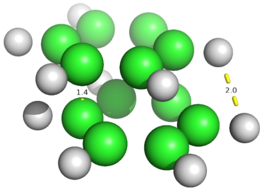	π-π stacking fulfilling rules

Table S8: Molecules specifically designed for π-π stacking tests.

**File name (pdb)**	**Image**	**Description**
VdW1	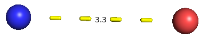	Two atoms fulfilling default rules for V.d.W bonds
VdW2	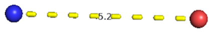	Two atoms not fulfilling default rules for V.d.W bonds
VdW3	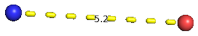	Two atoms not fulfilling default rules for V.d.W bonds
VdW4	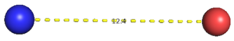	Two atoms not fulfilling default rules for V.d.W bonds
VdW5	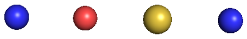	Four atoms where pairs do not fulfill default rules for V.d.W bonds
VdW6	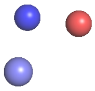	Three atoms, two of which satisfy the V.d.W. rules while one does not.
VdW7	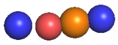	Four atoms that satisfy default V.d.W rules between them.

Table S9: Molecules specifically designed for VdW tests.

File name (pdb)	Image	Description
Picat1	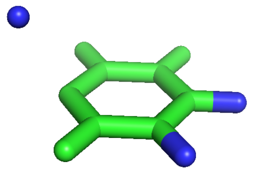	Aromatic ring from HIS and a cation (ND1) not fulfilling default rules for π-cation bond.
Picat2	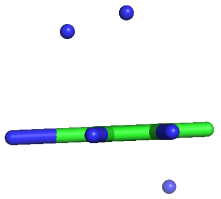	Aromatic ring from TYR and four cations (ND1).
Picat3	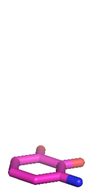	Aromatic ring from TYR and one cation (NZ) not fulfilling default parameters for π-cation bond.
Picat4	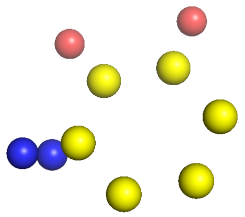	Aromatic ring from TYR and one cation (NZ).
Picat5	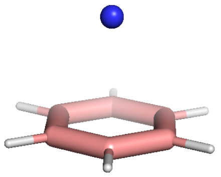	Aromatic ring from TYR and one cation (NZ).

Table S10: Molecules specifically designed for π-cation tests.

File name (pdb)	Image	Description
Hyd1	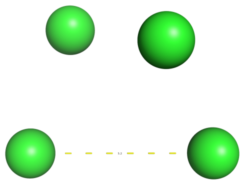	4 C_α chain with an assessed hydrophobic bond (ILE-LEU)
Hyd2	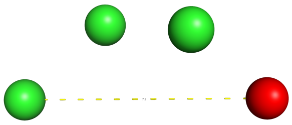	C_α chain without hydrophobic bond.
TrpHyd	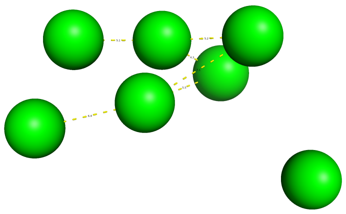	C_α backbone from Trp-Cage miniprotein.

Table S11: Molecules specifically designed for hydrophobic tests.

We proceed now by listing the various tests that have been performed. Each row of the following tables indicates the molecule and the input parameters used for the test, as well as the expected results. More precisely, Table S12 illustrates the tests relative to the ionic bond, Table S13 illustrates the H-bond tests, Table S14 shows the tests designed for π-π Stacking bonds, Table S15 lists the tests for VDW bonds, Table S16 shows the tests used for the $\pi-$cation bonds and Table S17 lists the tests for the hydrophobic bonds.

Test #	File name	Ionic Distance Param.	Expected ionic edges
1	IonIon1	default	0
2	IonIon2	1.23	2: (Lys1,Glu4),(His10,Asp7)
3		1.2	0
4	IonIon3	default	3: (Lys4,Asp1),(His10,Glu7),(Lys4,Glu7)
5		3.2	2: (Lys4,Asp1),(His10,Glu7)
6		1.8	1: (His10,Glu7)
7		1.59	0
8	IonIon4	default	1: (Glu1,Lys4)
9		2.54	0
10	IonIon5	default	2: (Glu1,Lys4),(Glu1,Arg7)
11		1.98	1: (Glu1,Lys4)
12		1.93	0
13	IonIon6	default	0
14	IonIon7	default	0
15	IonIon8	3.2	1: (Lys4,Asp1)
16		3.1	0
17	IonIon9	default	0

Table S12: Ionic tests and expected results

Test #	File name	H-bond Angle Param.	H-bond distance Param.	Expected H-bond edges
18	Hbond1	default	default	0
19	Hbond2	default	default	0
20	Hbond3	default	default	1: (Asp9,Arg2)
21		default	2	0
22		1.2	default	0
23	Hbond4	default	default	1: (Lys2,Gln9)
24		default	2.4	0
25		0.03	default	0
26	Hbond5	default	default	2: (Asn5,Asn1),(Asn5,Asn2)
27		default	2.02	1: (Asn5,Asn2)
28		default	2	0
29		0.0189	default	1: (Asn5,Asn2)
30		0.0186	default	0
31	Hbond6	default	default	3: (Lys2,Ser5),(Lys2,Ser8),(Lys2,Ser11)
32		default	2.026	2: (Lys2,Ser5),(Lys2,Ser11)
33		default	2.01	1: (Lys2,Ser5)
34		default	2	0
35		0.025	default	2: (Lys2,Ser8),(Lys2,Ser11)
36		0.02	default	1: (Lys2,Ser8)
37		0.012	default	0
38	Hbond7	default	default	1: (Lys2,Ser5)
39		default	2.01	0
40		0.012	default	0
41	Hbond8	default	default	1: (Lys2,Gln9)
42		default	2.55	0
43		0.64	default	0

Table S13: H-bond tests and expected results

Test #	File name	π-π Normal-center Angle Param.	π-π distance Param.	Expected π-π edges
44	PiPi1	default	default	0
45	PiPi2	default	default	0
46	PiPi3	default	default	0
47	PiPi4	default	default	0
48	PiPi5	default	default	1: (Phe1,Phe4)
49	PiPi6	default	default	1: (Phe1,Phe4)
50		45.0	default	0
51		default	1.9	0

Table S14: π-π tests and expected results

Test #	File name	VdW Distance Param.	Expected VdW edges
52	VdW1	default	1: (Asn1,Gln4)
53		3.2	1: (Asn1,Gln4)
54	VdW2	default	0
55	VdW3	default	0
56	VdW4	default	0
57	VdW5	default	0
58	VdW6	default	0
59	VdW7	default	1: (Gln4,Asn7)
60		0.48	0
61	VdW8	default	4: (Asn1,Asn4),(Gln7,Gln1),(Gln1,Asn4), (Gln7,Asn4)
62		0.37	3: (Gln7,Gln1),(Gln1,Asn4), (Gln7,Asn4)
63		0.34	2: (Gln1,Asn4), (Gln7,Asn4)
64		-1.59	1: (Gln7,Asn4)
65		-1.62	0

Table S15: VdW tests and expected results

Test #	File name	π-cation Distance Param.	Expected π-cation edges
66	Picat1	default	0
67	Picat2	default	4: (Tyr1,Lys4),(Tyr1,Lys6),(Tyr1,Lys7), (Tyr1,Lys8)
68		4.001	3: (Tyr1,Lys6),(Tyr1,Lys7),(Tyr1,Lys8),
69		3.9999	1: (Tyr1,Lys7)
70		3.9	0:
71	Picat3	default	0
72	Picat4	default	0
73	Picat5	default	1: (Tyr1,Lys4)
74		2.98	0

Table S16: π-cation tests and expected results

Test #	File name	Expected Hydrophobic edges
75	Hyd1	1: (Leu1,Ile4)
76	Hyd2	0
77	TrpHyd	6: (Ile4, Leu7), (Gly10,Leu7), (Gly11,Leu7), (Leu2,Trp6), (Gly10,Trp6); (Gly11,Trp6)

Table S17: Hydrophobic bond tests and expected results

References

[1] I. Bahar and R.L. Jernigan. Inter-residue potentials in globular proteins and the dominance of highly specific hydrophilic interactions at close separation. *Journal of Molecular Biology*, 266(1):195–214, 1997.

[2] Riccardo Chelli, Francesco Luigi Gervasio, Piero Procacci, and Vincenzo Schettino. Inter-residue and solvent-residue interactions in proteins: A statistical study on experimental structures. *Proteins: Structure, Function, and Bioinformatics*, 55(1):139–151, 2004.

[3] Blagoje P. Dimitrijević, Sunčica Z. Borozan, and Srđan Đ. Stojanović. $\pi-\pi$ and cation-π interactions in protein-porphyrin complex crystal structures. *RSC Adv.*, 2:12963–12972, 2012.

[4] Cedrix J. Dongmo Foumthuim, Manuel Carrer, Maurine Houvet, Tatjana Škrbić, Giuseppe Graziano, and Achille Giacometti. Can the roles of polar and non-polar moieties be reversed in non-polar solvents? *Phys. Chem. Chem. Phys.*, 22:25848–25858, 2020.

[5] Leonardo H. R. Dos Santos, Anna Krawczuk, and Piero Macchi. Distributed atomic polarizabilities of amino acids and their hydrogen-bonded aggregates. *The Journal of Physical Chemistry A*, 119(13):3285–3298, 2015. PMID: 25760575.

[6] Jacob N. Israelachvili. *Intermolecular and Surface Forces.* Academic Press Inc., New York, second edition edition, 1992.

[7] Jacob N. Israelachvili. *Intermolecular and Surface Forces.* Academic Press Inc., New York, third edition edition, 2011.

[8] Christoph Janiak. A critical account on $\pi - \pi$ stacking in metal complexes with aromatic nitrogen-containing ligands. *J. Chem. Soc., Dalton Trans.*, pages 3885–3896, 2000.

[9] William L. Jorgensen and Julian Tirado-Rives. The opls [optimized potentials for liquid simulations] potential functions for proteins, energy minimizations for crystals of cyclic peptides and crambin. *Journal of the American Chemical Society*, 110(6):1657–1666, 1988.

[10] Wolfgang Kabsch and Christian Sander. Dictionary of protein secondary structure: Pattern recognition of hydrogen-bonded and geometrical features. *Biopolymers*, 22(12):2577–2637, 1983.

[11] Georgia B McGaughey, Gagné Marc, and Anthony K. Rappé. π-stacking interactions. alive and well in proteins. *Journal of Biological Chemistry*, 273(25):15458–15463, 1998.

[12] Angelo Onofrio, Giovanni Parisi, Giuseppe Punzi, Simona Todisco, Maria Antonietta Di Noia, Fabrizio Bossis, Antonio Turi, Anna De Grassi, and Ciro Leonardo Pierri. Distance-dependent hydrophobic-hydrophobic contacts in protein folding simulations. *Phys. Chem. Chem. Phys.*, 16:18907–18917, 2014.

[13] Robert S. Paton and Jonathan M. Goodman. Hydrogen bonding and $\pi-$stacking: How reliable are force fields? a critical evaluation of force field descriptions of nonbonded interactions. *Journal of Chemical Information and Modeling*, 49(4):944–955, 2009.

[14] Ranjit Thakuria, Naba K. Nath, and Binoy K. Saha. The nature and applications of $\pi - \pi$ interactions: A perspective. *Crystal Growth & Design*, 19(2):523–528, 2019.

[15] Seiji Tsuzuki, Kazumasa Honda, Tadafumi Uchimaru, Masuhiro Mikami, and Kazutoshi Tanabe. Origin of attraction and directionality of the $\pi - \pi$ interaction: Model chemistry calculations of benzene dimer interaction. *Journal of the American Chemical Society*, 124(1):104–112, 2002.

[16] Aysegul Turupcu, Julian Tirado-Rives, and William L. Jorgensen. Explicit representation of cation$-\pi$ interactions in force fields with 1/r4 nonbonded terms. *Journal of Chemical Theory and Computation*, 16(11):7184–7194, 2020.

[17] Angelo Vedani. Yeti: An interactive molecular mechanics program for small-molecule protein complexes. *Journal of Computational Chemistry*, 9(3):269–280, 1988.

[18] M. Vijayakumar and Huan-Xiang Zhou. Prediction of residue-residue pair frequencies in proteins. *The Journal of Physical Chemistry B*, 104(41):9755–9764, 10 2000.

[19] Yuan Zhao, Jue Li, Hui Gu, Dongqing Wei, Yao-chang Xu, Wei Fu, and Zhengtian Yu. Conformational preferences of $\pi - \pi$ stacking between ligand and protein, analysis derived from crystal structure data geometric preference of $\pi - \pi$ interaction. *Interdisciplinary Sciences: Computational Life Sciences*, 7(1):211–220, 2015.
